# Prognostic and predictive value of AXL and C-MET in patients with rectal cancer

**DOI:** 10.1590/0102-67202025000049e1918

**Published:** 2026-01-19

**Authors:** Carmen Austrália Paredes Marcondes RIBAS, Efstathia N DOELKEN, Sudipta TRIPATHI, Bülent POLAT, Reinhard LISSNER, Thomas BÖELDICKE, Jurandir Marcondes RIBAS-FILHO, Osvaldo MALAFAIA, Martin GASSER, Ana Maria WAAGA-GASSER

**Affiliations:** 1 Faculdade Evangélica Mackenzie do Paraná – Curitiba (PR), Brazil.; 2 University of Wuerzburg, Molecular Oncology and Immunology, Department of Surgery I – Wuerzburg (BY), Germany.; 3 University of Massachusetts Chan Medical School, Department of Medicine, Division of Renal Medicine – Worcester (MA), USA.; 4 University of Wurzburg, University Hospital, Department of Radiation Oncology – Wurzburg (BY), Germany; 5 Helmholtz Centre for Infection Research – Braunschweig, Germany.; 6 Harvard Medical School, Brigham and Women’s Hospital, Renal Division, Department of Medicine – Boston (MA), USA.

**Keywords:** Rectal Cancer, Biomarkers, Protein-Tyrosine Kinases, Neoplastic Stem Cells, Chemoradiotherapy, Câncer Retal, Biomarcadores, Proteínas Tirosina Quinases, Células-Tronco Neoplásicas, Quimioradioterapia

## Abstract

**Background:**

Rectal cancer remains a significant clinical challenge with demand for conclusive biomarkers, essential in prognostication and therapy monitoring of neoadjuvant and adjuvant treatment strategies.

**Aims:**

The aim of the study was to evaluate AXL and cellular mesenchymal-epithelial transition factor (C-MET) biomarkers for cancer stem cells and to correlate them with clinicopathological characteristics and patient outcome data with respect to neoadjuvant chemoradiotherapy.

**Methods:**

Serum levels of soluble surface markers AXL and C-MET were retrospectively analyzed in 164 rectal cancer patients with additional immunofluorescent analyses of their primary tumor tissues.

**Results:**

Kaplan-Meier analysis confirmed the prognostic significance of Union for International Cancer Control stages, but with no significant correlation between investigated markers with patient age, gender, or tumor stage. In contrast, tumor tissues demonstrated stage-dependently increased marker expression. While AXL was detected at low levels, C-MET exhibited a bimodal distribution, with elevated levels seen in most patients, particularly post-neoadjuvant therapy and non-significantly in the subgroup with poorer response to neoadjuvant therapy (p=0.074).

**Conclusions:**

AXL serum levels in the rectal cancer cohort were significantly different from healthy subjects but did not correlate with tumor stage or survival during and after neoadjuvant/adjuvant therapy. Soluble C-MET levels in the blood, influenced by neoadjuvant chemoradiotherapy, may serve as a predictive marker for treatment response.

## ARTICLE HIGHLIGHTS

Rectal carcinoma remains a significant global health challenge due to the risk of developing the disease increasing steadily with advancing age.

Preoperative assessment of serum tumor markers provides valuable prognostic and therapeutic information in solid tumors.

Persistently elevated tumor markers, despite resection, suggest residual disease or not detectable micrometastases, which may guide decisions on adjuvant therapy.

Rectal cancer patients showed elevated C-MET levels in their serum, which were correlated with its expression in the respective patient tumors. Serum levels for AXL were found to be diminished.

Neoadjuvant chemoradiotherapy resulted in elevated C-MET levels, suggesting that it may serve as a predictive marker for treatment response.

## Summary

**CENTRAL MESSAGE:**

Rectal cancer remains a significant clinical challenge, with demand for conclusive biomarkers essential in prognostication and therapy monitoring of neoadjuvant and adjuvant treatment strategies. Serum levels of soluble surface markers AXL and C-MET, described for cancer stem cells in rectal cancer, possibly can correlate with clinicopathological characteristics and patient outcome data with respect to neoadjuvant chemoradiotherapy.

**PERSPECTIVES:**

AXL and C-MET serum levels in rectal cancer are significantly different from healthy subjects but did not correlate with tumor stage or survival during and after neoadjuvant/adjuvant therapy, while C-MET may hold promise for influencing future treatment responses.

## VISUAL ABSTRACT

**Figure:**
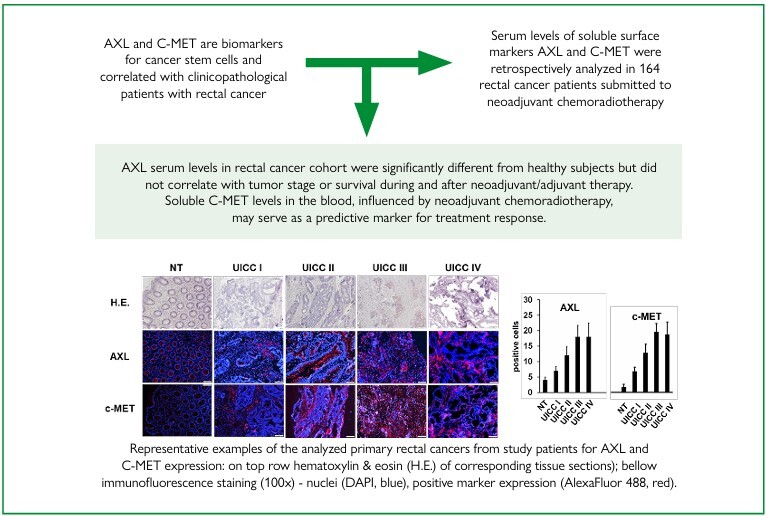

## RESUMO

**Racional:**

O câncer de reto continua sendo desafio clínico significativo com demanda por biomarcadores conclusivos, essenciais no prognóstico e monitoramento terapêutico de estratégias de tratamento neoadjuvante e adjuvante.

**Objetivos:**

Avaliar os biomarcadores AXL e C-MET para células-tronco cancerígenas e correlacioná-los com características clínico-patológicas e dados de resultados de pacientes em relação à radioquimioterapia neoadjuvante.

**Métodos:**

Os níveis séricos dos marcadores de superfície solúveis AXL e C-MET foram analisados retrospectivamente em 164 pacientes com câncer retal com análises adicionais de imunofluorescência de seus tecidos tumorais primários.

**Resultados:**

A análise de Kaplan-Meier confirmou a significância prognóstica dos estágios da UICC, mas sem correlação significativa entre os marcadores investigados com a idade, sexo ou estágio do tumor do paciente. Em contraste, os tecidos tumorais demonstraram expressão de marcadores aumentada em função do estágio. Enquanto a AXL foi detectada em níveis baixos, o C-MET exibiu distribuição bimodal, com níveis elevados observados na maioria dos pacientes, particularmente na terapia pós-neoadjuvante e de forma não significativa no subgrupo com pior resposta à terapia neoadjuvante (p=0,074, p>0.05).

**Conclusões:**

Os níveis séricos de AXL na coorte de câncer retal foram significativamente diferentes dos indivíduos saudáveis, mas não se correlacionaram com o estágio do tumor ou a sobrevida durante e após a terapia neoadjuvante/adjuvante. Os níveis de C-MET solúvel no sangue, influenciados pela radioquimioterapia neoadjuvante, podem servir como um marcador preditivo para a resposta ao tratamento.

## INTRODUCTION

 Rectal carcinoma remains a significant global health challenge due to the risk of developing the disease increasing steadily with advancing age. The 5-year survival rate for rectal cancer based on actual SEER (Surveillance, Epidemiology, and End Results) data from the American Cancer Society is only slightly higher than that for colon cancer (67 vs. 63%). However, stage-specific survival is almost the same for both tumors, although rectal cancer patients generally have a higher risk of locoregional recurrence than colon cancer patients and some studies also show a higher risk for systemic recurrence with spread to distant organs despite advances in multimodal treatment^14,18^. 

 Preoperative assessment of serum tumor markers provides valuable prognostic and therapeutic information in solid tumors. Although not diagnostic on their own, elevated preoperative levels often correlate with tumor burden, advanced stage, and adverse outcomes. Patients with high preoperative markers typically have worse outcomes (shorter disease-free and overall survival). They serve as baseline references for postoperative monitoring. Persistently elevated tumor markers, despite resection, suggest residual disease or not detectable micrometastases, which may guide decisions on adjuvant therapy^21^. They can be used to assess the success of the treatment and, if necessary, to detect a recurrence. Carcinoembryonic antigen (CEA) is an established prognostic factor in colorectal cancer (CRC), and is routinely determined preoperatively in affected patients^4,9^. In gastrointestinal malignancies, CEA together with the carbohydrate antigen 19-9 (CA19-9) contributes to risk stratification^9^, while in ovarian cancer, carbohydrate antigen CA-125 (CA-125) predicts disease stage and resectability. For testicular cancer, AFP (alpha-fetoprotein), β-hCG (beta-human chorionic gonadotropin), and LDH (lactate dehydrogenase) are essential for staging and treatment planning. Despite their limitations in specificity and sensitivity, preoperative serum tumor markers remain important tools to refine prognosis, guide adjuvant therapy decisions, and enable effective postoperative surveillance. In the era of modern neoadjuvant and adjuvant treatment strategies, there seems to be a high demand for such soluble marker profiles in the blood beyond standard CEA and CA19-9 determination in these patients^4,9^. 

 Cancer stem cells (CSCs) play a central role in the initiation and progression of tumors as well as tumor recurrences^9^. Another characteristic of this tumor cell population is its increased resistance to chemotherapy. This property is at least partly due to the high expression of drug transporters, which, for example, pump alkylating agents out of the cell, thereby preventing effective damage to tumor cells by chemotherapy. The subject of intense research over the last years is the identification of tumor stem cell-specific surface markers and molecular signaling pathways to distinguish tumor stem cells from normal cells, as well as from normal stem cells, and, if necessary, to recognize or eliminate them in a targeted manner. Several potential tumor stem cell markers were suggested to be linked to CRC^12,20^. 

 New CSC biomarkers could also enable an individualized assessment of the course of therapy. Membrane-bound tyrosine kinase receptors (RTK) such as AXL (receptor tyrosine kinase) perform central tasks in cell-cell communication. The stimulation of the PI3K (phosphatidylinositol-3 kinase) protein and its target molecule Akt (A group of enzymes involved in several processes related to cell growth and survival)is a central step in AXL-dependent signaling^12^. The stimulation of the P13K protein and its target molecule Akt is a central step in AXL-dependent signaling^12^. AXL is particularly overexpressed in highly malignant colon cancers and peritoneal metastases. Increased concentrations of AXL are associated with a significantly worse disease prognosis and shorter survival times. In addition, overexpression of AXL in tumor cells contributes to chemotherapy resistance and dramatically reduced survival rates^2^. AXL receptor activation occurs through ligand-dependent GAS6 (growth arrest-specific 6) driven mechanisms but also through crosstalk with other receptors like C-MET (cellular mesenchymal-epithelial transition factor) or EGFR (epidermal growth factor receptor)^22^. An increase in the migration and invasion potential of tumor cells has also been described in various adenocarcinomas, malignant melanomas, and squamous cell carcinomas. Moreover, AXL is a key driver of stemness in cancer cells, facilitating self-renewal and contributing to the long-term maintenance of tumors such as breast cancer. Its expression correlates with various cancer stem cell markers, including CD44 (cluster of differentiation 44) and ALDH1 (aldehyde dehydrogenase 1)^24^. These findings suggest that AXL may be a significant prognostic biomarker and a promising therapeutic target in CRC. Recent studies focused on its expression in both colon and rectal cancers together^16^, the majority of them not clearly taking into consideration the effects of neoadjuvant chemoradiotherapy on the rectal cancers in their studies, which may have a tremendous influence on molecular expression patterns. There had been particularly no studies regarding the expression of AXL as a suitable blood biomarker in rectal cancer patients throughout neoadjuvant treatment protocols, which was investigated for the first time in this study. 

 The hepatocyte growth factor (HGF) receptor C-MET also belongs to the family of tyrosine kinase receptors. In normal adult tissue, the protein is expressed only at low levels and mainly in cells of epithelial or mesenchymal origin. The expression of MET and its ligand HGF is associated with various biological processes, including embryogenesis, cell proliferation, cell survival, differentiation, and tissue repair. Excessive expression of the protein is closely related to cancer-promoting processes^6^. Various signaling pathways are involved in further signal transduction by C-MET, such as the PI3K/AKT (phosphatidylinositol-3 kinase/serine/threonine protein kinase), the RAS/RAF/MEK/ERK (rat sarcoma virus/rapidly accelerated fibrossarcoma/Mitogen-activated protein kinase kinase/extracellular signal-regulated kinases), and the SRC/FAK (encodes a non-receptor tyrosine kinase/focal adhesion kinase) signaling pathways. Activation of these molecular cascades induces cell growth, proliferation, survival, cell motility, and angiogenesis^19^. C-MET appears to play a significant role in tumorigenesis. Overexpression of C-MET has been observed in breast, bronchial, gastric, kidney, and colon cancers^6^. In CRC in particular, overexpression of C-MET is associated with advanced tumor stages, appears to stimulate VEGF-A (vascular endothelial growth fator-A) production and thus angiogenesis in tumor cells, and promotes therapy resistance in patients treated with anti-EGFR monoclonal antibodies^3^. It has been linked to aggressive tumor phenotypes and poorer clinical outcomes. Targeting C-MET signaling may overcome resistance to conventional treatments. 

 AXL and C-MET serve as important markers of tumor stem cell activity in CRC, providing prognostic information related to tumor aggressiveness, metastatic potential, and therapeutic resistance. Their expression levels may guide clinical decision-making, and they represent potential targets for novel therapeutic interventions aimed at improving patient outcomes. Further research is warranted to validate their prognostic utility and to develop effective CSC-targeted therapies in rectal cancer^15,20^. 

 Rectal cancers exhibit distinct molecular signatures compared to colon cancer, particularly regarding gene mutation profiles, chromosomal instability (CIN), transcriptomic expression, and molecular subtypes. Rectal cancers are enriched for TP53 (tumor protein 53) and NRAS (neuroblastoma RAS viral oncogene homolog) mutations and demonstrate a higher prevalence of the CIN pathway^7^. C-MET is influenced by NRAS mutations, as NRAS-mutated melanomas, for instance, show higher activation of the C-MET pathway and increased sensitivity to C-MET inhibitors compared to BRAF (human gene that encodes a protein called B-Raf) mutated or wild-type melanomas. NRAS mutations appear to uniquely impact C-MET phosphorylation (i.e., activation) and downstream effects on cell proliferation and migration in response to its ligand, HGF. However, this relationship between NRAS gene mutations and C-MET expression was not yet observed for CRCs, presumably because of analyzing CRCs without distinguishing and focusing on those that overexpress NRAS, such as in rectal cancer. Moreover, rectal cancers can now be classified into distinct molecular subtypes, such as the RSS1 (sucrose synthase 1)and RSS2 (sucrose synthase 2) subtypes, which are distinct from the broader consensus molecular subtypes used for general CRC^13^. These molecular differences underscore the distinct biological mechanisms underlying rectal and colon cancer, influencing treatment approaches and prognostic predictions. 

 This study focused on the expression and diagnostic/prognostic potential of the two tyrosine kinases, AXL and C-MET, in rectal cancer, individually or in combination. For this purpose, serum levels were determined to retrospectively investigate whether predictions about the course of the disease and long-term patient survival could have been derived from these. 

## METHODS

### Patients

 In this study, data were collected retrospectively from 164 patients (42 females and 122 males) with histologically confirmed rectal cancer treated at the University Hospital of Wuerzburg, Germany, and were evaluated for this study. Neoadjuvant therapy of rectal cancer patients consisted of either short-term radiotherapy or long-term chemoradiotherapy: short-term radiation was performed with 5×5 Gy/day up to 25 Gy of total radiation dose over a week of the primary rectal tumor and surrounding tissue within the pelvis. Long-term chemoradiotherapy was carried out with 28×1.8 Gy up to a total dose of 50.4 Gy over 6 weeks with additional chemotherapy (two cycles of 5-fluorouracil [5FU] with 100 mg/m^2^ body surface/day 1–5 in week 1 plus 5, or a combination of 5-FU 250 mg/m^2^ day 1–14 + 2235 with oxaliplatin with 50 mg/m^2^ day 1, 8, 22, and 29). The primary tumor therapy of these patients was carried out exclusively at the Surgical Department at the University Hospital in Wuerzburg, Germany. After discharge from the clinic, patients were seen in the Surgical Polyclinic or affiliated oncological centers or outpatient clinics of the Comprehensive Cancer Center at the University Hospital as part of their tumor aftercare, and further data were collected. The follow-up included a physical examination, blood sampling, chest X-ray, abdominal ultrasound, and computed tomography or magnetic resonance imaging, depending on the guidelines of the German tumor centers. For this purpose, the parameters to be examined were evaluated using data from files recorded by the International Classification of Diseases key. The data collection included the following essential parameters: gender, age, and tumor stage, as classified by the UICC (Union for International Cancer Control) system. The samples to be examined were obtained after full consent of the patients from the Tumor Tissue and Sera Bank from The Interdisciplinary Bank and Data Wuerzburg at the University Hospital Wuerzburg^10^. The blood samples were taken by the Department of Radio-Oncology at the University Hospital before and during chemoradiotherapy, the Surgical Clinic before the operation, as well as at outpatient oncology centers thereafter, and analyzed^10^. The study was approved by the Ethics Committee of the Institution. 

### Serum determination of C-MET and AXL by ELISA

 Serum from the blood samples was analyzed for C-MET and AXL using commercial Enzyme-Linked Immunosorbent Assay (ELISA) kits according to the protocols specified by the respective manufacturers. C-MET Protein (Invitrogen, human c-Met [soluble] ELISA Kit, Catalog # KHO2031, serum: 1:100), AXL (Abnova, AXL human ELISA Kit, Catalog # SEE824, serum: 1:100). The sera of a total of 39 healthy subjects were used as controls. The assays were performed according to the manufacturer’s instructions. Analysis was carried out in duplicates. The results were read by an ELISA reader (Dynatech Laboratories, Sullyfield, USA) at 450 nm and are expressed in ng/mL or pg/mL. 

### Immunofluorescent staining

 Primary antibodies against C-MET (EPR19067.1:1000 diluted, ab216574) and AXL (EPR238.1:500 diluted, ab259831) were purchased from Abcam (Cambridge, UK). Secondary Cy3 (indocarbocyanine) conjugated and AlexaFluor 488 conjugated antibodies were obtained from Jackson ImmunoResearch (West Grove, PA, USA). The staining was performed on cryostat sections of snap-frozen tumor specimens. Samples were fixed in acetone, permeabilized in methanol, and incubated with the primary antibody in TBS (tris buffered saline) plus 0.5% bovine serum albumin overnight at 4°C in a humidified chamber. Treatment with the secondary fluorochrome-conjugated antibody was performed for 30 min at room temperature in a humidified chamber. Subsequently, slides were analyzed using an Olympus BX51 microscope and the CellSens Dimension software. Two independent investigators analyzed tumor tissue sections using a quantitative interpretation assessment (mean number of positively stained cells in three consecutively analyzed highpower fields). In the case of divergent results, mutual consent was obtained. 

### Statistical analysis

 GraphPad Prism 6 was used to create the figures and the associated statistical analyses. Additionally, the statistics program SPSS 12.0 from the University of Wuerzburg’s Computer Center was used. p≤0.05 were considered significant (*). Values for p<0.01 were considered highly significant (**). To test the data for statistically significant differences, the following tests were used in this work: chi-square test — based on this univariate analysis, the relationship between the two different proteins C-MET and AXL was analyzed; unpaired t-test; one-way ANOVA; Kaplan-Meier; and log-rank test. Tumor-related survival describes the period between the time of diagnosis and death/last contact with the patient. Recurrence-free period describes the period between diagnosis and the first recurrence. Censored data were defined and used when the event (tumor-related death/recurrence) had not yet occurred by a certain time (last seen).. 

## RESULTS

### Gender, age distribution, and tumor stage according to Union for International Cancer Control

 Of the 164 patients with rectal cancer that were included in this study, a threefold difference in gender distribution, with predominantly male compared to female individuals, was evident (122/164, 74.4% vs. 42/164, 25.6%, Table 1). Due to the significantly higher number of male patients and the insufficient number of female patients for a separate evaluation, all patients were included in the assessment, regardless of their gender. The patients enrolled were between 31.4 and 83.6 years old, with a median age of 64.3 years. The median follow-up was 45 months, and 53/164 patients (32.3%) had died of their tumor. About one third of the patients (37.2%) were in an early tumor stage (UICC 0 and 1) with Tis/T1/T2, N0, and M0 at the time of diagnosis, while just under 15% of the patients (n=24) already had distant metastases and thus a late stage prevailed. 

**Table 1:** Patient distribution based on Union for International Cancer Control stage.

		Patients	
		(n)	(%)
Tumor stage			
	T1	12	7.3
	T2	50	30.5
	T3	80	48.8
	T4	6	3.7
	Tx	16	9.8
Lymph node status			
	N0	104	63.4
	N1	35	21.3
	N2	22	13.4
	Nx	3	1.8
Metastasis status			
	M	134	81.7
	M1	24	14.6
	Mx	6	3.7
UICC post-op			
	UICC 0	14	8.5
	UICC I	47	28.7
	UICC II	41	25
	UICC III	38	23.2
	UICC IV	24	14.6

UICC: Union for International Cancer Control.

### Influence of prognostic factors on tumor-related survival

**Figure 1:**
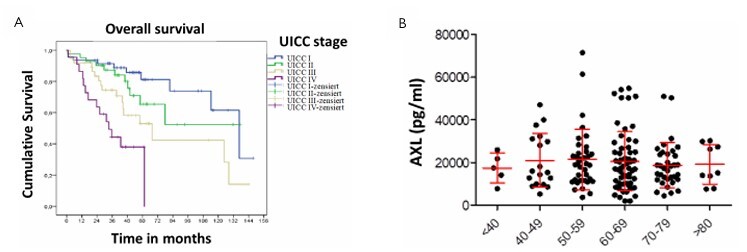
Influence of the Union for International Cancer Control stage on overall survival in patients with rectal cancer (Kaplan-Meier curve). (A) correlation of the AXL serum levels with age (categorized in age groups) (B) data for C-MET not shown.

**Figure 2:**
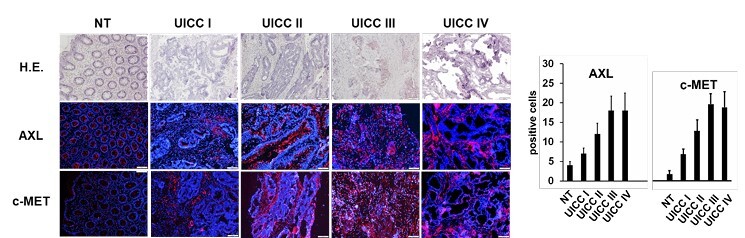
Representative examples of the analyzed primary rectal cancers from study patients for AXL and C-MET expression: on top row, hematoxylin and eosin (H.E.) of corresponding tissue sections; below immunofluorescence staining (100×) — nuclei (DAPI ((4’,6-diamidino-2-phenylindole) blue), positive marker expression (AlexaFluor 488, red).

 The influence of relevant clinical and pathological factors in the rectal cancer patients was first investigated using the Kaplan-Meier method. There was no significant influence of gender and age of the patients on tumor-related survival. In contrast, a highly significant impact of the UICC stage on the survival of patients was observed (p<0.001, Figure 1A), which underlines the prognostic importance of the UICC classification and the relevance of the studied patient cohort. Immunofluorescence analysis of primary tumors from analyzed patients showed increased expression for the two investigated markers, particularly in advanced stages of the tumor (UICC stages III and IV), compared to early stages (UICC stages I and II), and based on low expression levels in normal rectal tissue (Figure 2). This upregulated expression was particularly observed for both AXL and C-MET. 

### Correlation of AXL and C-MET serum levels with patient-relevant factors and tumor characteristics

 For AXL, higher values were observed in men compared to women. However, the differences for AXL did not reach statistical significance (p=0.06; two-sided t-test). For C-MET, no significant correlation was found (data not shown). 

**Figure 3:**
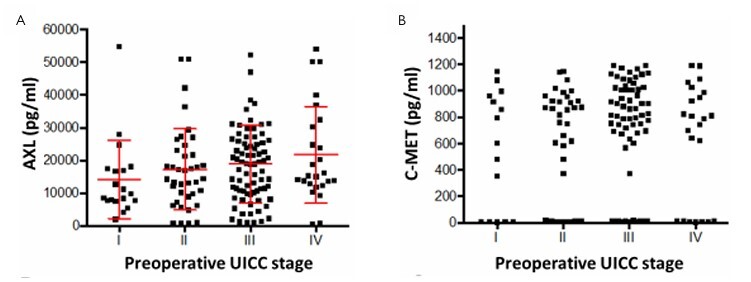
Comparison of AXL (A) and C-MET (B) serum levels with Union for International Cancer Control stages of the patients.

 Subsequently, we investigated whether the serum levels of the two parameters were associated with the age of the patients. There was no significant correlation between AXL or between the expression of C-MET serum levels (data not shown) and the different age groups of the patients. Interestingly, none of the markers demonstrated a significant association with the UICC stage of the patients (Figure 3). 

### Correlation between AXL and C-MET with overall patient survival and disease-free survival

**Figure 4:**
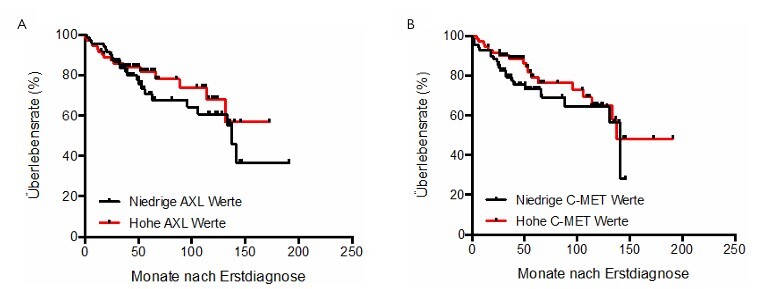
Comparison of AXL (A) and C-MET (B) serum levels with patient survival.

**Figure 5:**
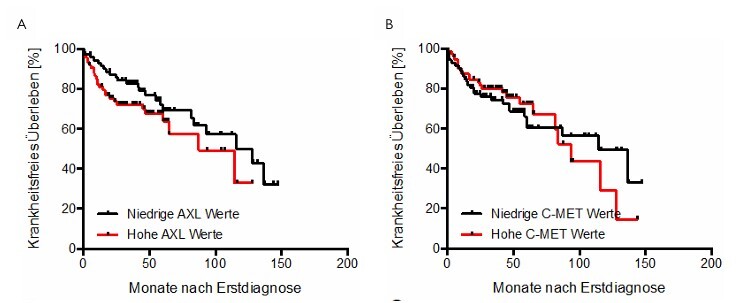
Comparison of AXL (A) and C-MET (B) serum levels with patient disease-free survival.

 Using the Kaplan-Meier method, the influence of serum expression levels of AXL and C-MET on both overall survival and disease-free survival (DFS) of the patients was investigated. There was no significant influence on the overall survival (Figure 4) or on DFS (Figure 5) of the patients. 

### Correlation of serum levels of AXL and C-MET in patients with rectal cancer and healthy volunteers, with the influence of the therapy

**Figure 6:**
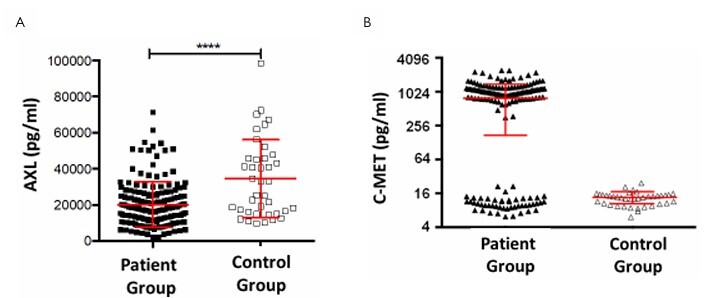
Correlation of AXL (A) and C-MET (B) serum levels of the tumor patients and healthy volunteers with the influence of the therapy.

 The significance of the serum levels of AXL and C-MET was assessed in all rectal cancer patients and compared with the group of healthy individuals (n=39). For AXL, significantly lower serum levels were found in the rectal cancer patients (p<0.0001, two-sided unpaired t-test, mean: 34.5 vs. 20.2 ng/mL in the controls (Figure 6A, 6B). Surprisingly, the evaluation of C-MET levels revealed two different patient subgroups. While more than two-thirds of the patients (111/159=70%) demonstrated higher C-MET values than healthy controls, onethird (48 patients=30%) showed, on average, almost 100-fold lower values. For better visualization of the two groups, the measured values for C-MET are shown in a log scale format. 

 To clarify whether the reduced AXL levels in the serum of the patients could be explained either by the tumor down-regulating this tyrosine kinase receptor AXL or by intervention through surgery or neoadjuvant radiation therapy in these patients, the correlation between the time of blood sampling relative to the operation was examined. 

 The patients were divided into a total of five groups:

1. Blood sampling more than 2 weeks before surgery;
2. Within 2 weeks before surgery;
3. On the day of surgery (before the start of the tumor resection);
4. Within 2 weeks after surgery; and
5. More than 2 weeks after surgery. Interestingly, again significantly lower AXL serum values were found in the tumor patients compared to healthy controls.

**Figure 7:**
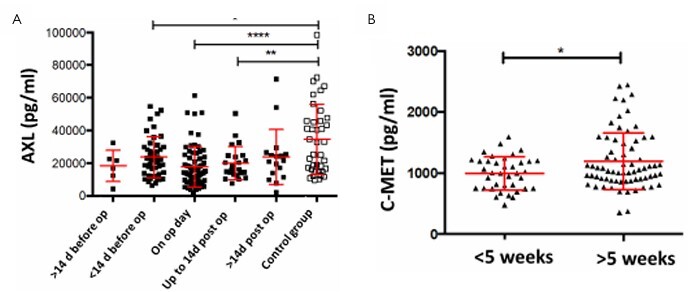
Correlation of AXL (A) serum levels at different time intervals before and after removal of the tumor (healthy volunteers for comparison). The patients were divided into five groups: (i) blood sampling more than 2 weeks before surgery, (ii) within 2 weeks before surgery, (iii) on the day of surgery (before the start of the removal of the tumor), (iv) within 2 weeks after surgery, and (v) more than 2 weeks after surgery and compared with healthy volunteers. (B) The influence of neoadjuvant chemoradiotherapy on the expression of C-MET. The patients were divided into two groups: (i) those who had received short-term radiotherapy (1 to ≤5 weeks of neoadjuvant therapy) and (ii) those who had undergone long-term chemoradiotherapy (≥5 weeks).

**Figure 8:**
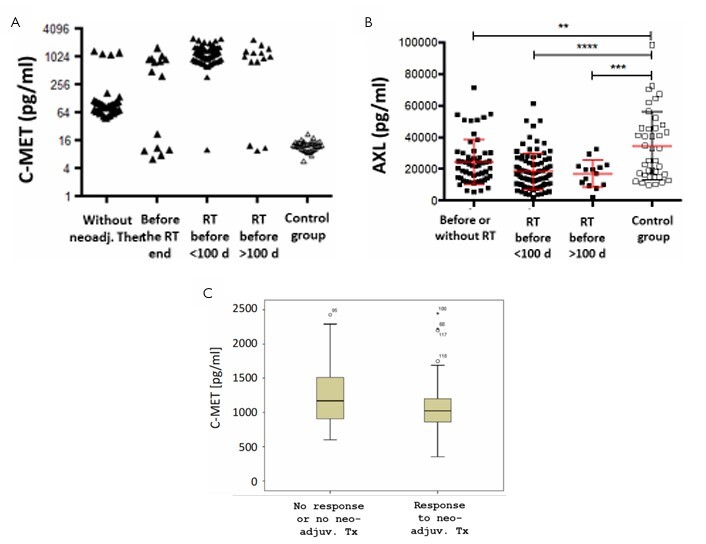
Correlation of AXL (A) and C-MET (B) serum levels of the tumor patients and healthy volunteers with the influence of the therapy. Patients with high C-MET values showed a rather poorer response to neoadjuvant therapy (C).

However, this was independent of the time of the operation (Figure 7A). The removal of the tumor during the surgery obviously did not influence the AXL serum levels (Figure 7B). Interestingly, low-level C-MET values such as in healthy controls occurred almost exclusively in patients without or before radiation therapy (data not shown), while highly elevated CMET levels occurred after radiation therapy (Figure 8A). 

 The influence of the neoadjuvant therapy in rectal cancer patients on AXL levels was then further determined. AXL values were found to be always decreased in rectal cancer patients with and without accomplished neoadjuvant radiation therapy and, also, independent of the time after radiation therapy and surgery (Figure 8B). In contrast, for C-MET, with a clear correlation with neoadjuvant radiation therapy, patients without prior therapy usually had low C-MET levels. In contrast, high values were detectable in pre-treated patients, suggestive of upregulated intracellular pathways involving C-MET in rectal tumor patients, which are involved in tissue repair, including cell proliferation. Patients with high C-MET values showed a rather poorer response to neoadjuvant therapy (Figure 8C), although the corresponding p-value of 0.074 did not reach statistical significance and was also influenced by the subgroup of patients with no radiation therapy that was included in this subgroup analysis. 

## DISCUSSION

 In this study, we analyzed serum expression levels of the two potential biomarkers, AXL and C-MET, in a cohort of 164 patients with newly diagnosed rectal carcinoma. Our analysis aimed to clarify their association with clinico-pathological characteristics, overall survival, DFS, and response to neoadjuvant chemoradiotherapy. 

 Consistent with epidemiological data, our cohort showed a male predominance in rectal cancer incidence, with a median age of 64.3 years, reflecting typical demographic patterns reported in prior studies. The tumor stages at diagnosis were distributed across early and advanced stages, with approximately 15% of patients presenting with distant metastases. Kaplan-Meier analysis confirmed strong prognostic significance of the UICC stage, supporting its continued use as a cornerstone of rectal cancer prognostication. Interestingly, neither of the two serum markers demonstrated a significant association with patient age, gender, or tumor stage. AXL showed a non-significant trend toward higher expression in men. The lack of correlation between serum marker levels and tumor stage suggests that systemic levels of these proteins may not directly reflect tumor burden in rectal cancer, unlike their tissue expression, which was notably increased in advanced tumors of the patients herein studied. This divergence between tissue and serum levels is consistent with previous studies, suggesting that serum biomarker levels are influenced by additional factors, such as clearance, shedding, or therapy-induced modulation^5,8^. 

 The analysis of survival outcomes revealed no significant association of AXL or C-MET with overall survival or DFS. Indeed, high serum levels seem to be associated with shorter survival rates and higher recurrence rates in several tumors^1,2,6,11^. This finding suggests that these serum markers alone may have limited prognostic value for survival in rectal cancer patients, although their role in conjunction with other markers or in specific subgroups cannot be excluded. 

 Among the two potential markers that are described to be expressed by tumor cells and also CSCs, only C-MET showed a potential influence on response to neoadjuvant therapy, with higher pre-treatment levels associated with poorer response. While the observed trend did not reach statistical significance (p=0.074), likely due to sample size limitations and inclusion of patients without neoadjuvant therapy, it highlights the potential of C-MET as a predictive marker for chemoradiotherapy response. While patients without neoadjuvant chemoradiotherapy had low serum levels compared to controls, preirradiated patients, i.e., after radiotherapy and before surgery, showed elevated serum levels, depending on the dose of radio-/chemoradiotherapy. Thus, a low C-MET level could induce the expression of genes (including C-MET) that are involved in tissue repair and regeneration due to its tissue-damaging effect. The reduced serum values in tumor patients without chemoradiotherapy should therefore be followed up and reproduced and investigated in independent measurements as well as in a second independent cohort including patients with other gastrointestinal malignant diseases. 

 Serum levels of AXL were significantly lower in rectal cancer patients. This observation is in congruence with the overexpression observed in other tumor cells^11,17,23^. It implies potentially tumor-mediated dysregulation of these markers, independent of surgical tumor removal or neoadjuvant therapy, as levels remained stable before and after surgery. For C-MET, a bimodal pattern emerged: roughly two-thirds of patients had elevated levels, while one-third exhibited markedly reduced levels. Notably, elevated C-MET levels correlated with prior neoadjuvant radiation therapy and were further influenced by the duration of radiation exposure, suggesting radiationinduced upregulation of C-MET-related pathways involved in tissue repair or proliferation of cells. Whether proliferation of cells may also comprise radio-/chemoresistant tumor cells with CSC characteristics expressing C-MET remains questionable and would demand further investigation of C-MET-mediated proliferation of such cells^15^. 

 Overall, our findings suggest that AXL expression in the serum may serve as a biomarker for rectal cancer presence, while C-MET shows potential utility as a marker for therapy response and radiation-induced tissue changes. Further studies with larger, treatment-stratified cohorts are highly warranted to validate these findings and further clarify their prognostic and predictive role, and moreover, the mechanistic basis of serum marker expression changes. Moreover, longitudinal monitoring of these markers could provide insights into tumor biology, treatment effects, and tissue repair processes in rectal cancer patients. 

 This study is limited by its retrospective design, the uneven gender distribution, and the relatively small control cohort. The heterogeneity of study cohorts through neoadjuvant chemoradiotherapy versus no such therapy in some patients and the timing of blood sampling as shown influences serum marker levels and should be addressed in future prospective studies. Despite growing evidence for the prognostic relevance of these markers, particularly for C-MET, challenges remain before their routine clinical application. Standardization of detection methods, cut-off thresholds, and validation in large, prospective cohorts with marker evaluation over more than 200 days during neoadjuvant and adjuvant therapies and surgical therapy seems to be necessary. Moreover, the interplay between these markers and their combined prognostic value warrants further exploration. 

## CONCLUSIONS

 Serum levels for AXL were found to be diminished. Interestingly, these findings were not associated with tumor stage or survival, which was in contrast to their protein expression in the tumor tissues in this study and already published data by others. More interestingly, rectal cancer patients showed elevated C-MET levels in their serum, which was correlated with its expression in the respective patient tumors. Neoadjuvant chemoradiotherapy resulted in elevated C-MET levels suggestive to serve as a predictive marker for treatment response. Although not statistically significant because of limited numbers and in part influenced by patients who got no neoadjuvant therapy in this study, those with a response to therapy showed lower C-MET levels. This supports published data from primary tumor analyses by others and the potential value of C-MET for defining response to therapy. These findings underscore the complexity of using serum biomarkers, particularly in patients with rectal cancer undergoing neoadjuvant and adjuvant chemoradiotherapy, and highlight C-MET as a candidate for further investigation in predictive and response monitoring contexts. 

## Data Availability

The datasets generated and/or analyzed during the current study are available from the corresponding author upon reasonable request.
